# Supplementary material for: Evaluation of bacterial proliferation with a microfluidic-based device: Antibiochip
Source: PLoS One. 2020 Feb 27;15(2):e0223932. doi: 10.1371/journal.pone.0223932 (PMC7046301; doi:10.1371/journal.pone.0223932)
Supplement: Attachment — Submitted filename: response_to_Reviewers_Antibiochip.docx [file pone.0223932.s001.docx]

Dear Dr. Shamala Devi Sekaran,

thank you for considering our paper “Evaluation of bacterial proliferation with a microfluidic-based device: Antibiochip.” worth evaluating. We are submitting a new version of the paper where we tried to address all the points highlighted by the reviewers.

In the following an explanation to specific questions is reported. On the website we will upload also, as required:

- A marked-up copy of the manuscript that highlights changes made to the original version
- An unmarked version of the revised paper without tracked changes.
- The additional figures

Best regards

Marco Pizzi

1. When submitting your revision, we need you to address these additional requirements.
Please ensure that your manuscript meets PLOS ONE's style requirements, including those for file naming. The PLOS ONE style templates can be found at
<http://www.journals.plos.org/plosone/s/file?id=wjVg/PLOSOne_formatting_sample_main_body.pdf> and <http://www.journals.plos.org/plosone/s/file?id=ba62/PLOSOne_formatting_sample_title_authors_affiliations.pdf>

Reply:

Certainly, we will do it

2. We note that 'The microorganisms used in this study were E. coli isolated from clinical samples'. Please include the reference if the clinical study that the E coli samples were isolated from has already been published.

Reply:

Used clinical samples are not coming from clinical studies. Indeed, the very large majority of microbiology tests are performed in out-patients not involved in controlled clinical trials.

3. Thank you for stating the following in the Financial Disclosure section:

The author(s) received no specific funding for this work.

We note that one or more of the authors are employed by a commercial company: "ELTEK S.p.A." and "Phenomix S.r.l."

1. Please provide an amended Funding Statement declaring this commercial affiliation, as well as a statement regarding the Role of Funders in your study. If the funding organization did not play a role in the study design, data collection and analysis, decision to publish, or preparation of the manuscript and only provided financial support in the form of authors' salaries and/or research materials, please review your statements relating to the author contributions, and ensure you have specifically and accurately indicated the role(s) that these authors had in your study. You can update author roles in the Author Contributions section of the online submission form.

Please also include the following statement within your amended Funding Statement.

“The funder provided support in the form of salaries for authors [insert relevant initials], but did not have any additional role in the study design, data collection and analysis, decision to publish, or preparation of the manuscript. The specific roles of these authors are articulated in the ‘author contributions’ section.”

If your commercial affiliation did play a role in your study, please state and explain this role within your updated Funding Statement.

Reply:

MP, VG, MZ and PB are employees of ELTEK S.p.A and developed the micro-channels and the centrifuge in their plant, with the support of ELTEK funding.

GM is a co-founder and scientific director of Phenomix ltd, AR is a biotechnologist working as research assistant in Phenomix ltd. Phenomix was partially granted by ELTEK as research contractor and partially supported the study with reagents, materials and human resources.

SL and TP are employees of a private laboratory, belonging to a multinational organization that allowed the use of its microbiology unit for studies of the prototypes with human pathogens. No other funding or support was available.

We can confirm that “The funder provided support in the form of salaries for authors [MP, VG, MZ and PB], but did not have any additional role in the study design, data collection and analysis, decision to publish, or preparation of the manuscript. The specific roles of these authors are articulated in the ‘author contributions’ section.”

2. Please also provide an updated Competing Interests Statement declaring this commercial affiliation along with any other relevant declarations relating to employment, consultancy, patents, products in development, or marketed products, etc.

Within your Competing Interests Statement, please confirm that this commercial affiliation does not alter your adherence to all PLOS ONE policies on sharing data and materials by including the following statement: "This does not alter our adherence to  PLOS ONE policies on sharing data and materials.” (as detailed online in our guide for authors <http://journals.plos.org/plosone/s/competing-interests>) . If this adherence statement is not accurate and  there are restrictions on sharing of data and/or materials, please state these. Please note that we cannot proceed with consideration of your article until this information has been declared.

Please include both an updated Funding Statement and Competing Interests Statement in your cover letter. We will change the online submission form on your behalf.

Please know it is PLOS ONE policy for corresponding authors to declare, on behalf of all authors, all potential competing interests for the purposes of transparency. PLOS defines a competing interest as anything that interferes with, or could reasonably be perceived as interfering with, the full and objective presentation, peer review, editorial decision-making, or publication of research or non-research articles submitted to one of the journals. Competing interests can be financial or non-financial, professional, or personal. Competing interests can arise in relationship to an organization or another person. Please follow this link to our website for more details on competing interests: <http://journals.plos.org/plosone/s/competing-interests>

Reply:

We confirm that our affiliation does not alter our adherence to PLOS ONE policies on sharing data and materials.

MP, VG, MZ and PB as employees of ELTEK S.p.A, founder of the research, performed the research as part of their normal job activity, so no conflict is foreseen. For GM, AR, TP and SL, no competing interests at all as well.

4. PLOS requires an ORCID iD for the corresponding author in Editorial Manager on papers submitted after December 6th, 2016. Please ensure that you have an ORCID iD and that it is validated in Editorial Manager. To do this, go to ‘Update my Information’ (in the upper left-hand corner of the main menu), and click on the Fetch/Validate link next to the ORCID field. This will take you to the ORCID site and allow you to create a new iD or authenticate a pre-existing iD in Editorial Manager. Please see the following video for instructions on linking an ORCID iD to your Editorial Manager account: <https://www.youtube.com/watch?v=_xcclfuvtxQ>

Reply:

Ok, done.

Additional Editor Comments:

Please refer to the comments by both reviewers. The writing of the methods and the discussion require extensive rewriting and to be relevant to the study. Methodology needs to be clear.


**Comments to the Author**

1. Is the manuscript technically sound, and do the data support the conclusions?

The manuscript must describe a technically sound piece of scientific research with data that supports the conclusions. Experiments must have been conducted rigorously, with appropriate controls, replication, and sample sizes. The conclusions must be drawn appropriately based on the data presented.

Reviewer #1: Partly

Reviewer #2: No

2. Has the statistical analysis been performed appropriately and rigorously?

Reviewer #1: No

Reviewer #2: No

3. Have the authors made all data underlying the findings in their manuscript fully available?

The [PLOS Data policy](http://www.plosone.org/static/policies.action#sharing) requires authors to make all data underlying the findings described in their manuscript fully available without restriction, with rare exception (please refer to the Data Availability Statement in the manuscript PDF file). The data should be provided as part of the manuscript or its supporting information, or deposited to a public repository. For example, in addition to summary statistics, the data points behind means, medians and variance measures should be available. If there are restrictions on publicly sharing data—e.g. participant privacy or use of data from a third party—those must be specified.

Reviewer #1: Yes

Reviewer #2: No

4. Is the manuscript presented in an intelligible fashion and written in standard English?

PLOS ONE does not copyedit accepted manuscripts, so the language in submitted articles must be clear, correct, and unambiguous. Any typographical or grammatical errors should be corrected at revision, so please note any specific errors here.

Reviewer #1: No

Reviewer #2: Yes

5. Review Comments to the Author

Please use the space provided to explain your answers to the questions above. You may also include additional comments for the author, including concerns about dual publication, research ethics, or publication ethics. (Please upload your review as an attachment if it exceeds 20,000 characters)

Reviewer #1: Although the technology has clinical impact especially in determining antibiotic susceptibility, the identification of microorganisms still needs the normal culturing Technics. The only advantage is the duration of AST result reporting is shortened, however, this is a very preliminary data and need validation at least with minimum 5 microorganisms with respective standard drugs.

Reply:

Being the centrifuge and the micro-channels (the main added values of the method) prototypes non already commercially available, we of course can publish the protocol but in the absence of this tools, researchers could have problems in reproducing the experiments.

What it should be done could be the installation of the tools in another microbiology lab, where other microbiologists could reproduce the experiments. This is a really unusual procedure in applied research but it can be done. In any case we are working in a further evolution of the system with the objective to increase the user friendliness and accordingly allowing an easier testing in other labs.

Following are my detailed comments:
Methodology:

In detail, the bacterial strains used in this study were obtained from a clinical microbiology laboratory studying samples from hospital and community infections.- Pls indicate how many strains used.

Reply:

OK. A table with the list of microbes was added to the manuscript.

Measurement of microbial concentration by VSMCs.- The bacterial suspension was used when the McFarland number was 1 or more – This is not clear. Pls indicate in CFU/ml

Reply:

We calculated the CFU/mL corresponding to 1 McFarland. In a series of experiments, it resulted to be 0.5 x 10^9^ E. coli/mL

Pls change centrifugation force units from RPM to g.

Reply:

Ok. See the revised version of the manuscript.

In a first series of tests, two-fold dilutions of each microbe were loaded into the microchannel prechamber,and when the microchannels were filled with the suspension, disks containing the VSMCs were centrifuged at 7000 rpm for 3 minutes, according to the operating conditions identified in the preclinical (industrial feasibility) phase of the study. During this set of experiments, different microbes were plated in VSMCs at different angles and volumes.- Pls indicate what microbes are these.

Reply:

OK: In all tests, virtually only Enterobacteriaceae (Gram – bacteria) were used.

Another series of tests was carried out to evaluate whether VSMCs were suitable to detect not only the differences in microorganism concentrations obtained by a serial dilution but also the inhibition of bacterial proliferation caused by antibiotics in susceptible strains. For this, different microbes were incubated in a plastic tube at 37°C in a moist chamber in the presence of the MIC of different antibiotics- Same here , pls indicate list of microbes with respective concentration (cfu.ml) used.

After 30 and 60 minutes, a microsample of the culture was collected by a micropipette and loaded into the loading chamber of the VSMC. – what is the volume used here?

Reply:

See the revised version of the manuscript

Results –

No statistical analysis were performed for data presented in Fig 3,4, 5,6,7,8,

Reply:

A simple statistical analysis is present in these figures, represented by mean and the error bars. However, additional statistical analysis has been added to the manuscript.

Fig 8- implicates that anything below 40% inhibition is resistant, pls explain if this cut-off value is applicable for all bacterial microorganisms. Otherwise indicate that this is applicable only for E coli.

Reply:

OK. See the revised version of the manuscript

Discussion:

Overall the results implicate that although the technology/method is novel but it is still at very preliminary stage. The identification of bacteria (genus and species) is still not detectable using this method. This should be further elaborated as drawback of this device.

Reply:

This is an interesting point raised by the reviewer. However, this set of experiments was carried out in order to really speed the TAT of AST, in particular because for ID, novel and rapid methods (such as Maldi TOF) are now available at diagnostic level.

Reviewer #2: 1.Abstract is adequate
2. Introduction - too brief, needs more elaboration

Reply:

OK. See the revised version of the manuscript

3. Methods: The details of the procedure not included, i.e: starting concentration of the organism, procedure of performing, media used, growth condition, antibiotic susceptibility testing results, etc.

Reply:

OK. See the revised version of the manuscript

4. Results: How do you compare inhibition rate? The susceptibility of organism is not shown.
The growth curve of the organism is not measured. Therefore, there is no baseline to compare proliferation inhibition rate.

Reply:

OK. See the revised version of the manuscript; in particular, the susceptibility of different microbes to ATB using standard tests is shown in Table 2. The inhibition is calculated respect to the growth on the microbe without ATB in 60 minutes.

Growth rate in 1 hour is extremely difficult to be performed, in particular using classic methods. The VSMC method seems, at least in our knowledge, the first attempt to measure the proliferation in a very short period of time

5. Discussion: The discussion is not relevant i.e the comparison with resistant stains (this data was not shown by other methods in the methods and results)

Reply:

Resistant strains were originally identified by routine test. The discussion was deeply revised to answer the reviewer’s observation.

6. PLOS authors have the option to publish the peer review history of their article ([what does this mean?](https://journals.plos.org/plosone/s/editorial-and-peer-review-process#loc-peer-review-history)). If published, this will include your full peer review and any attached files.


If you choose “no”, your identity will remain anonymous but your review may still be made public.

Reply:

Not relevant for us.

**Do you want your identity to be public for this peer review?** For information about this choice, including consent withdrawal, please see our [Privacy Policy](https://www.plos.org/privacy-policy).

Reviewer #1: No

Reviewer #2: No
